# Alcoholization as an Alternative Treatment for Prostatic Cyst and Abscess in Dogs

**DOI:** 10.3390/ani15121818

**Published:** 2025-06-19

**Authors:** Giulia Ballotta, Giuseppe Spinella, Marco Cunto, Daniele Zambelli

**Affiliations:** Department of Veterinary Medical Sciences, University of Bologna, 40064 Bologna, Italy; giulia.ballotta2@unibo.it (G.B.); giuseppe.spinella@unibo.it (G.S.); daniele.zambelli@unibo.it (D.Z.)

**Keywords:** prostate, prostatic cyst, prostatic abscess, dog, alcoholization, ethanol, alcohol sclerotherapy, percutaneous ethanol injection (PEI)

## Abstract

Current therapeutic approaches for prostatic cysts and abscesses involve surgical methods like reduction and omentalization. This retrospective study evaluates the effectiveness and safety of ultrasound-guided percutaneous drainage combined with alcoholization as a minimally invasive treatment for prostatic cysts and abscesses in dogs. Forty-three male dogs with a single prostatic cavity (1–6 cm) were enrolled and categorized into two groups: group A (twenty-two dogs with prostatic abscesses treated via alcoholization) and group B (twenty-one dogs with BPH-associated cysts). Group B was further subdivided into three subgroups: B1 (finasteride only), B2 (alcoholization only), and B3 (combined finasteride and alcoholization). Alcoholization involved sedation, drainage of the cavity, and two ethanol instillations (first: 1/3–1/4 of aspirated volume; second: 1/10 after 25 min). Ultrasound evaluations were performed at scheduled intervals of up to 150 days. In group A, 76.19% of abscesses were resolved within 30 days, with complete resolution in all dogs by day 150. In group B, the combined treatment (group B3) showed superior outcomes, with significant cyst diameter reduction observed in all dogs within 30 days and complete resolution in 62.5% by day 150. These findings support alcoholization as an effective, low-complication alternative to surgery for selected prostatic lesions in dogs.

## Simple Summary

Abnormalities of the canine prostate gland, such as benign hyperplasia, cysts, and prostatic abscess, are common in old male dogs. Nowadays, the most common treatment strategies for prostatic cysts and abscesses, where feasible, include surgical approaches such as drainage, reduction and omentalization. This retrospective study describes the treatment of prostatic cysts and abscesses via ultrasound-guided percutaneous drainage and alcoholization, with the aim of contributing to the limited number of cases present in the literature regarding this technique, providing our experience and detailing the procedure, outcomes, and potential side effects. The results of this study highlight alcoholization as a minimally invasive alternative to traditional surgical methods for managing a single small prostatic cavity, well surrounded by prostatic parenchyma and not communicating with the urethra. Its low complication rate makes it a viable option for select cases.

## 1. Introduction

Intact male dogs over 5 years of age are frequently involved in benign prostatic hyperplasia (BPH) [1,2,3,4]. Although most dogs with BPH remain asymptomatic [1], some may exhibit prostatic fluid discharge from the prepuce, hematospermia, and occasionally microhematuria or hematuria when sanguineous discharges reach the urinary bladder. Additionally, due to dorsal outgrowth of the prostatic gland in dogs, clinical signs such as rectal tenesmus with small, thin, tape-shaped feces; constipation; dyschezia; and perineal hernias may occur [1,2,5,6]. Less commonly, dysuria or urinary incontinence may be observed with increasing prostate volume or when large cysts are present [7,8,9,10]. Dogs with BPH are predisposed to the development of prostatic cysts and prostatitis, which may progress to abscess formation [5]. Prostatic cysts generally result from degenerative changes in the glandular parenchyma that lead to obstruction of the excretory ducts, accumulation of prostatic fluid, and subsequent cyst formation during BPH progression [1,4,11,12]. Cavitary formations such as cysts and abscesses can be treated with different surgical techniques [13,14,15], minimally invasive techniques [16], or conservative treatments, including percutaneous drainage alone [17,18] or in combination with intracavitary agents such as absolute ethanol (alcoholization technique or percutaneous ethanol injection—PEI or alcohol sclerotherapy) [17,19,20,21] or platelet-rich plasma (PRP) [22] or tea tree oil [23]. Hormonal therapies, including the administration of progestogens or antiandrogens (osaterone acetate) [24,25,26] or 5-alpha-reductase inhibitors (finasteride) [2,27,28] and GnRH analogs (deslorelin acetate) [29,30], have also been used. In human medicine, Espinoza reported that parenchymal intraprostatic injection of ethanol in 60 men with a mean age of 66.5 years was well tolerated and led to a mean prostatic volume reduction of approximately 35% [31]. In veterinary medicine, Abu-Seida et al. (2012) [20] demonstrated the efficacy of the alcoholization technique in dogs with BPH by parenchymal intraprostatic injection of ethanol or oxytetracycline HCl under ultrasound guidance. Ethanol resulted in more effective treatment than oxytetracycline, with nearly normal-sized prostates in all patients after two months post-injection [20]. More recently, this technique has been successfully applied as sclerotherapy for cystic or abscess formations in various organs and viscera, such as the liver, thyroid or kidney [32,33,34]. However, ultrasound-guided percutaneous drainage followed by alcoholization of the prostatic cavity, a technique first described in 1999 [17], remains poorly documented in the literature. The aim of this study is to contribute to the limited number of cases present in the literature regarding this technique by reporting authors’ personal experiences and detailing the procedure, outcomes, and potential side effects of alcoholization (alcohol sclerotherapy) for the treatment of prostatic cysts and abscesses in dogs.

## 2. Materials and Methods

This study was based on a retrospective review of medical records of patients submitted to ethanol injection after a diagnosis of prostatic cysts and abscesses referred to the Department of Veterinary Medical Sciences between 2003 and 2023. The study was conducted in accordance with European Union Directive 2010/63/EU. The Animal Welfare Body of the University of Bologna provided a positive ethical and scientific opinion on the publication of the data, certifying that the study did not involve animal experimentation but rather clinical veterinary practice (prot. 143182/2025).

Forty-three dogs of various breeds, aged between 5 and 10 years and weighing between 15 and 20 kg, were retrospectively included in this study. Of these 43 patients, 21 had a prostatic abscess (group A), and 22 presented BPH associated with a single cystic formation (group B). Only dogs with abscesses or single cysts located within the prostatic parenchyma, well surrounded by the parenchyma, with a diameter ranging between 1 and 6 cm and not communicating with the urethra, were included in this study. Measurements of the largest diameter of the cystic or abscessual cavity and the entire surface area of the largest prostatic section were performed by ultrasound.

For both groups, the diagnosis was based on the patient history as well as clinical and ultrasound examinations and bacteriological, cytological, and biochemical analyses of the cavity fluid. Cytological and biochemical analyses were performed on the cavity fluid to exclude communication between the prostatic cavity and the urinary system. In particular, the absence of spermatozoa in the cytological samples, along with low levels of creatinine, urea, and potassium, comparable to those measured in blood, confirmed the absence of urinary contamination.

The selection of the therapeutic protocol for each dog—be it alcoholization alone, in combination with pharmacological agents, or pharmacological agents alone—was guided by the underlying pathology and additional considerations, such as the animal’s temperament, reproductive status, and situations in which the owner declined the recommended anesthetic protocol.

All twenty-one dogs included in group A were treated with alcoholization of the abscess in association with antimicrobial therapy. Patients were sedated with medetomidine (15 µg/kg I.M.), and ultrasound-guided percutaneous aspiration of purulent fluid was performed using 18–20 G spinal needles. An amount of absolute alcohol equal to 1/3–1/4 of the previously aspirated purulent fluid was then introduced into the residual cavity. The alcohol was then re-aspirated after 25 min and replaced with another ethanol injection equal to 1/10 of the volume of fluid initially aspirated. The antibiotic therapy was chosen based on antibiogram results performed on the abscess fluid collected on the day of diagnosis, and in all cases consisted of marbofloxacin (3 mg/kg SID), administered for 25 days. Ultrasound follow-ups were then performed at 0–5–30–150 days after alcoholization, measuring the dimensions of the residual cavity.

Twenty-two dogs included in group B, based on clinical records and therapeutic protocol, were assigned to three different subgroups: group B1—finasteride only (1.25 mg/subject/day, regardless of the dog’s size and severity of the disease, administered orally for 195 days [27]) (7/22); group B2—alcoholization only, performed using the same procedure as group A (7/22); group B3—combined therapy with finasteride (1.25 mg/subject/day, regardless of the dog’s size and severity of the disease, administered orally for 195 days [27]) and alcoholization, the latter performed 60 days after the start of finasteride treatment (8/22). The ultrasound checks were performed at 0–30–60–90–150 days after the start of therapy. In subgroup B3 dogs, the cyst diameter was assessed on day 60 of follow-up, prior to alcoholization treatment.

Cystic sizes were reported as mean and standard deviation (SD). One-way ANOVA was used to compare variations in diameters of cysts and prostatic cavities among the three subgroups of group B. Significance was set for *p* < 0.05.

## 3. Results

Recorded clinical signs of dogs included in group A on the day of the first visit (T0) were hyperthermia (body temperature ranged between 39.8 °C and 40.5 °C), sensorium depression, inappetence, polydipsia, and, in two cases, rectal tenesmus. In all dogs, blood tests revealed signs of infection, including elevated white blood cell count (WBC), increased neutrophils, lymphocytosis, and high C-reactive protein (CRP). Cytological examination of the abscess fluid showed numerous active granulocytes, the presence of free bacteria, some red blood cells, and rare prostatic cells showing signs of damage. Bacteriological cultures tested positive for bacteria sensitive to marbofloxacin: *Escherichia coli* (16/21), *Pseudomonas aeruginosa* (3/21) and *Staphylococcus* spp. (2/21). The volume of purulent material collected from all abscesses under ultrasound guidance ranged from 4 to 34.2 cc the day of treatment. A gradual improvement in signs until complete disappearance was observed within 3 days after alcohol intracavity injection. In six dogs (1, 5, 11, 13, 14 and 17), the abscess cavity was reduced until it disappeared five days after alcoholization, while in the remaining patients, a reduction in cavity diameter was also observed during the same period. After 30 days, only subjects 4, 7, 14 and 18 had a residual cavity (diameter 0.8, 0.6, 0.7, 0.6 and 0.9 cm, respectively), and, after 150 days, no cavity was visualized in all twenty-one included dogs. The diameter of abscess cavities assessed during ultrasound follow-ups in dogs from group A is reported in Table 1.

Moreover, no abscess recurrence was reported one year after treatment, and no side effects related to abscess alcoholization were observed.

In all dogs belonging to group B, blood tests showed no signs of infection and were within normal limits. Bacteriological cultures of the cystic fluid were negative for bacterial growth. Cytological examination of the cavity fluid revealed the presence of several to numerous polymorphonuclear neutrophilic granulocytes with highly segmented nuclei, along with macrophages, red blood cells, and rare clusters of mildly damaged prostatic cells. These findings are consistent with chronic inflammation.

Outcomes related to group B reported that the aspirated liquid from the cysts of groups B2 and B3 had a total volume ranging between 0.7 and 2.8 cc. No aspiration was performed in group B1. Symptoms described in clinical records were urethral blood loss and, in some cases, hematuria. All signs gradually improved until they disappeared in one week following the administration of finasteride therapy (groups B1 and B3). In group B2, the symptoms did not reveal any improvement. A significant reduction in the cavities’ diameters and of prostatic area was observed for groups B1, B2 and B3, between day 0 and day 150 (Table 2). However, following alcoholization, a more pronounced reduction was noted by day 60, and by day 150 a reduction of 90.33% was observed.

**Figure 1:**
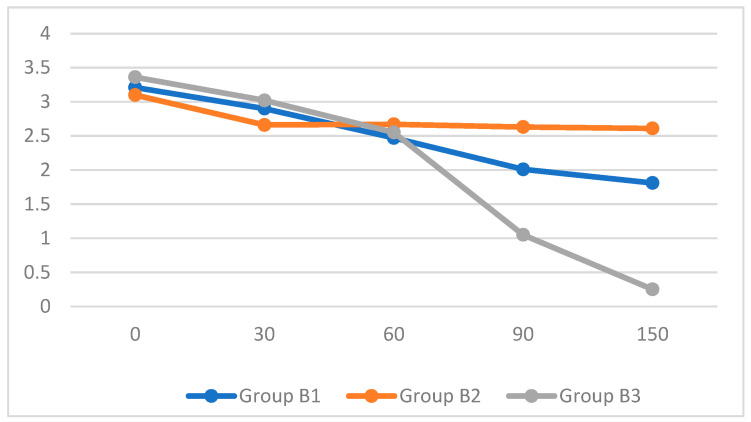
Diameters of the prostate cysts assessed at 0–30–60–90–150 days after treatment between the 3 subgroups. In the abscissa, the diameters of the cysts are given, whereas in the ordinate, the days of treatment are. Group B1: dogs treated with finasteride only; group B2: dogs treated with alcoholization only; group B3: dogs treated with a combination of alcoholization and finasteride.

In all subjects treated solely with finasteride (group B1), a reduction in cyst diameter was recorded from the first follow-up at 30 days of therapy. Specifically, between day 1 and day 150, a decrease of 43.55% was observed. However, in all seven subjects, the cyst was still present on day 150 of the therapy. Only in 1/7 of the subjects in group B2 was no reduction in cyst diameter observed; in the remaining subjects, a slight reduction of 15.67% of the initial diameter was noted between day 0 and day 150. Finally, in the eight subjects treated with both finasteride and alcoholization, a reduction in prostate cavity diameter was observed from the first follow-up at 30 days, almost identical to that of group B1. In 5/8 (62.5%) of the subjects at day 150, the prostatic cyst disappears. Figure 1 shows the diameters of the prostate cysts in the three subgroups at the various follow-up visits.

Using the one-way ANOVA test, significant differences in cyst diameter reduction in groups 2 and 3 were observed at days 90 and 150 (*p* < 0.05 and *p* < 0.01, respectively) (see Table 2). No side effects resulted from the ethanol injection.

## 4. Discussion

This study was intended to investigate the effects of percutaneous ethanol injection as treatment of single prostatic abscesses or cysts in dogs affected by benign prostatic hyperplasia with a diameter ranging between 1 and 6 cm. The therapeutic protocol applied for the prostatic abscess treatment (group A) demonstrated excellent outcomes, with complete healing and no complications or side effects during the procedure. These findings are consistent with those previously reported by Bussadori et al. in 1999 and Zambelli et al. in 2003 [17,21]. In 16 out of 21 cases, the abscess cavity had disappeared 30 days after treatment, while regression was achieved within 150 days after alcoholization in the remaining five cases, with no recurrence.

The results obtained in our study regarding the resolution of the pathology (infection) are similar to those described by Kawakami et al. [23], who used a percutaneous injection of tea tree oil alone without the addition of antimicrobial agents. However, we achieved complete resolution of the abscess, with the disappearance of the cavity, after a single ethanol injection combined with antimicrobial therapy in 76% (16/21) of cases at 30 days and in 100% of the patients by 150 days. In contrast, Kawasami et al. reported only a resolution of the infection and a reduction in prostatic cavity diameter after two injections of tea tree oil [23]. Certainly, the use of tea tree oil as an in situ treatment for prostatic abscesses represents a valid alternative to antimicrobial agents, especially considering the growing problem of antibiotic resistance. However, it only led to a reduction in the prostatic cavity, and although no recurrence was observed after one year, the possibility of reinfection remains. Conversely, the therapeutic approach described in this paper, combining PEI with antimicrobial therapy, resulted in complete resolution not only of the infection but also of the prostatic cavity.

Moreover, comparing the results obtained through the therapeutic approach here described with those achieved using antimicrobial treatment alone, it is observed that our protocol, which combines alcoholization with antimicrobial therapy, led to a complete and lasting resolution of the prostatic abscess without recurrences. In fact, in 2022, Lea et al. [35] conducted a retrospective study on 82 dogs affected by prostatitis and prostatic abscessation. Abscesses smaller than 1 cm were treated only with antimicrobial therapy, while abscesses larger than 1 cm were managed with ultrasound-guided drainage or omentalization, often associated with castration, with a 12% recurrence rate. Authors also reported that antimicrobial therapy lasted between 10 days and more than six months, with an average duration of 4–6 weeks [35]. In our study the duration of antimicrobial treatment, associated with PEI, was similar or shorter (25 days); notably, none of our patients experienced recurrence or underwent castration, thereby preserving their reproductive status, an important aspect for breeding animals. In relation to applied antimicrobial therapy, in both papers, fluoroquinolones were the most commonly prescribed drugs [35].

The use of alcoholization in the resolution of prostate cysts allowed us to collect different data between the three groups of animals. Administration of finasteride alone (group B1) allows it to act indirectly on cysts. This molecule, even if not registered in the EU for animals (Union Product Base: https://www.medicinesinfo.eu/select-language?destination=/node/210934, accessed on 1 January 2025), tends to reduce BPH, which has been shown to be the most frequent cause of cyst formation. Any reduction in diameter, associated with the disappearance of symptomatology, could be explained by the decrease in prostatic hyperplasia and the consequent reduction in compression on the glandular ducts and retention of fluid [20]. In fact, the azasteroid finasteride acts on type-II 5α-reductase, and it may decrease the prostatic volume up to 43%, as reported in a study by Sirinarumitr et al. [2], and almost similar to what was observed in the present study. Furthermore, it is known that short-term finasteride treatment (~2 months) is able to reduce overall effects of BPH, representing a valid therapy for BPH [28]. Mechanistically, finasteride reduces dihydrotestosterone (DHT) levels, induces apoptosis, and decreases tissue vascularity, contributing to prostate shrinkage and symptom relief. Conversely, alcoholization has been widely involved in the treatment of benign cysts in different organs (liver, kidney, thyroid, ovary, etc.) because of its induced effect of scleropathy, secondary to dehydration, protein denaturation, coagulative necrosis, and reactive fibrosis [34]. However, the administration of alcohol alone (group B2) seems to act mainly on the cysts and not on the surrounding prostatic parenchyma, which limits the overall therapeutic efficacy of this technique. This limitation may lead to cystic refilling, as BPH is not fully controlled by alcohol sclerotherapy alone. Studies on parenchymal intraprostatic ethanol injection indicate that ethanol’s effects are more pronounced when broadly targeting prostatic tissue rather than isolated cystic structures [20]. Conversely, combining finasteride with ethanol injection (group B3) yielded superior outcomes compared to either treatment alone (groups B1 and B2), confirming that combining treatments that address both the cysts and the hyperplastic prostatic tissue should be necessary for optimal results. Healing times observed in our study (significant reduction within 3–5 months for prostatic cysts in combination with finasteride) were in line with outcomes reported for renal cysts with lower ethanol concentration (83%) [33]. In fact, in 2023, Yoon et al. observed a cystic volume rate reduction from 86% to 90% between two and six months after treatment [33].

Moreover, the different outcome observed between group A (abscess treatment) and group B, as well as the limited efficacy of percutaneous ethanol injection alone for prostatic cysts (subgroup B2), can, in the authors’ opinion, be partly explained by the nature of the inner surface of the cystic cavity. This surface consists of a part of a glandular gland or duct with epithelial cell coating that could provide a sort of resistance to the rapid cytotoxic effect of alcohol. This may also explain why platelet-rich plasma (PRP) treatment for prostatic cysts in dogs, as described by Bigliardi et al. [22], yielded better results compared to PEI alone. In their study, prostatic cysts were no longer detectable by ultrasound at 24 and 60 days post-PRP injection in all ten dogs enrolled in their study. Conversely, in our study, complete resolution was observed in only 62.5% of dogs in subgroup B3 (finasteride associated with PEI). The superior efficacy of PRP may be attributed to its biological properties: PRP stimulates cell replication, modulates cellular differentiation, promotes angiogenesis and extracellular matrix synthesis, exerts significant antibacterial activity, and modulates the inflammatory response [22,36,37].

Although PRP can be considered, given its less aggressive nature and high efficacy, an excellent therapeutic strategy for the treatment of prostatic cysts in dogs, its application in routine clinical practice remains limited due to the difficulty in obtaining PRP. Therefore, since absolute ethanol is inexpensive and readily available, PEI represents a valid alternative, as it is a safe and efficient technique. No complications or side effects were observed during or after the procedure in our study, partly due to the method of administration. For PEI to be effective, ethanol must remain in contact with the cyst. To achieve this, after draining the cystic cavity, an equivalent volume of ethanol can be injected and left in situ to be gradually absorbed by the cyst wall. However, for large cysts, the volume of ethanol required may risk systemic intoxication upon absorption. To mitigate this, we injected a reduced volume of ethanol, approximately one-third to one-quarter of the drained volume, and allowed it to act in contact with the cyst wall for 25 min. After this period, the cavity was drained again, and a smaller volume of ethanol was reinjected and left in place. Although it would have been possible to leave a small residual volume of ethanol, complete aspiration was performed, which also removed necrotic debris resulting from the coagulative necrosis induced by ethanol contact. The volume of ethanol reinjected after 25 min was sufficient to continue the therapeutic effect while remaining safe for the patient when left in situ. In conclusion, despite the obvious limitations related to the retrospective nature of the study and the number of dogs included in this study, the alcoholization technique was found to be effective in the treatment of prostatic abscesses and in the volumetric reduction in prostatic cysts in association with finasteride when the cavitary formation appears small, with a single cavity, subcapsular and well surrounded by prostatic parenchyma.

In 2003, Boland et al. [38] described the technique of ultrasound-guided percutaneous drainage followed by antibiotic therapy and castration in 13 dogs with abscesses (n = 8) and cysts (n = 5). The results of this approach demonstrated safety and effectiveness as the one reported in the present study. However, the percutaneous ethanol injection described in the present study appears to offer faster resolution, fewer interventions, and preservation of reproductive function, especially in breeding animals. Boland et al.’s study supports the value of repeated drainage and antibiotic therapy but requires more procedures and routinely includes castration, potentially limiting its applicability in certain canine populations. Furthermore, the present study provides insights into the pathophysiology of cystic formations, emphasizing the need to treat not only the cavity but also the hyperplastic prostatic tissue. The combined use of finasteride and ethanol (group B3) appears to mirror the multifactorial origin of cysts, achieving more lasting outcomes.

Finally, based on the results obtained in this study, ultrasonographic percutaneous drainage combined with alcoholization may be considered a therapeutic approach for multiple prostatic cavities that are not in communication with the urinary system. However, certain precautions must be taken into account: the cavity should be well surrounded by prostatic parenchyma, and the number and size of the cysts must be accurately evaluated, as these factors can influence the duration and feasibility of the treatment, which is typically performed under sedation.

## 5. Conclusions

In conclusion, even if some limitations remain due to the retrospective nature of the study, the number of subjects included, and the lack of data on the actual volume of the prostatic cavities, the alcoholization technique has proven effective in the treatment of prostatic abscesses and in reducing the volume of prostatic cysts, especially when combined with finasteride. This is particularly true when the cavitary formation is small, single, subcapsular, and well-surrounded by prostatic parenchyma. No complications or side effects were observed during or after the procedure, and complete resolution of the abscess was achieved without recurrence. The duration of antimicrobial therapy, when necessary, was similar to or shorter than traditional protocols. Moreover, the alcoholization of the prostatic cavity permits the preservation of the reproductive status of the animals, a very important aspect for breeding animals.

Finally, because the prostatic cavities are not spherical structures, taking into account the volume rather than just the largest diameter could provide a more accurate assessment of therapeutic success. Unfortunately, due to the retrospective nature of the study, volumetric data were not recorded.

## Figures and Tables

**Table 1:** Diameters of abscess cavities assessed at 0–5–30–150 days after treatment in dogs included in group A.

Dog	Cavity Diameter * T0	Cavity Size [cm]		
		After 5 Days	After 30 Days	After 150 Days
1	3.2	0	0	0
2	5.0	2.5	0	0
3	4.3	2.9	0	0
4	4.5	1.2	0.8	0
5	3.9	0	0	0
6	4.8	0.9	0	0
7	5.8	1.4	0.6	0
8	3.2	0.9	0	0
9	2.8	1.0	0.7	0
10	4.6	1.0	0	0
11	3.7	0	0	0
12	3.9	0.5	0	0
13	4.1	0	0	0
14	3.7	0	0.6	0
15	5.2	1.8	0	0
16	3.3	0.8	0	0
17	2.9	0	0	0
18	5.1	2.1	0.9	0
19	4.6	1.3	0	0
20	3.4	0.5	0	0
21	5.8	2.3	0	0

* largest transverse diameter of the prostatic cavity.

**Table 2:** Diameter of the prostatic cyst assessed at 0–30–60–90–150 days after treatment and prostatic area between the three subgroups: group B1: dogs treated with finasteride only; group B2: dogs treated with alcoholization only; group B3: dogs treated with a combination of alcoholization and finasteride.

	Diameter of Cyst [cm]					Prostatic Area [cm^2^]	
	Day 0	Day 30	Day 60	Day 90	Day 150	Day 0	Day 150
Group B1 (*n* = 7) Mean ± SD	3.21 ± 1.05	2.9 ± 1.15	2.47 ± 1.12	2.01 ± 1.23	1.81 ± 1.14 *	24.04 ± 5.69	14.6 ± 4.07 *
Group B2 (*n* = 7) Mean ± SD	3.1 ± 1.09	2.66 ± 1.42	2.67 ± 1.43	2.63 ± 1.47 ^#^	2.61 ± 1.55 *^#^	26.29 ± 6.01	28.8 ± 3.53 *^#^
Group B3 (*n* = 8) Mean ± SD	3.63 ± 1.28	3.02 ± 1.22	2.55 ± 1.05	1.05 ± 0.75 ^#^	0.32 ± 0.62 *^#^	22.04 ± 6.98	15.33 ± 4.56 *^#^

* significant difference from day 0 between all subgroups. ^#^ significant difference between groups 2 and 3. SD = standard deviation.

## Data Availability

The original contributions presented in this study are included in the article. Further inquiries can be directed to the corresponding author.
